# Serum amyloid a as a high-resolution indicator of mucosal healing in ulcerative colitis

**DOI:** 10.1093/crocol/otag097

**Published:** 2026-09-01

**Authors:** Kotaro Akita, Yoshihiro Yokoyama, Yuta Shimomori, Tomoe Kazama, Masanori Nojima, Hiroshi Nakase

**Affiliations:** Division of Gastroenterology and Hepatology, Department of Internal Medicine, Sapporo Medical University School of Medicine, Sapporo, Hokkaido, Japan; Division of Gastroenterology and Hepatology, Department of Internal Medicine, Sapporo Medical University School of Medicine, Sapporo, Hokkaido, Japan; Division of Gastroenterology and Hepatology, Department of Internal Medicine, Sapporo Medical University School of Medicine, Sapporo, Hokkaido, Japan; Division of Gastroenterology and Hepatology, Department of Internal Medicine, Sapporo Medical University School of Medicine, Sapporo, Hokkaido, Japan; Center for Translational Research, The Institute of Medical Science Hospital, The University of Tokyo, Tokyo, Japan; Division of Gastroenterology and Hepatology, Department of Internal Medicine, Sapporo Medical University School of Medicine, Sapporo, Hokkaido, Japan

**Keywords:** ulcerative colitis, SAA, IL-1β, Mayo Endoscopic Subscore, MES 0-1

## Abstract

**Background:**

Endoscopy is the standard for assessing mucosal activity in ulcerative colitis (UC), but reliable biomarkers are needed. This study evaluated serum amyloid A (SAA) as a potential biomarker by comparing it with established markers in UC.

**Methods:**

We retrospectively evaluated 182 patients with UC who underwent blood and stool testing within 14 days of colonoscopy at a tertiary care hospital between April 2017 and January 2025. Patients were classified into 4 groups according to the Mayo Endoscopic Subscore (MES). Baseline characteristics and laboratory and endoscopic variables were compared across groups.

**Results:**

Serum SAA levels demonstrated strong positive correlations with leucine-rich α-2-glycoprotein (LRG) (*r* = 0.752) and C-reactive protein (CRP) (*r* = 0.717). Significant associations were also observed between SAA and MES, Mayo score, partial Mayo score, and Ulcerative Colitis Endoscopic Index of Severity (UCEIS) (all *P* < .01). SAA identified active endoscopic inflammation (MES ≥ 2) at a cutoff value of 4.1 mg/L, with an area under the curve (AUC) of 0.813 (95% CI, 0.731-0.894), sensitivity of 0.804, and specificity of 0.741. In addition, SAA differentiated MES 0 from MES 1 at a cutoff value of 2.6 mg/L, yielding an AUC of 0.683 (95% CI, 0.470-0.897), sensitivity of 0.706, and specificity of 0.714.

**Conclusions:**

SAA exhibits diagnostic performance comparable to, and in some respects exceeding, that of established inflammatory biomarkers in UC. Importantly, SAA effectively discriminates between MES 0 and MES 1, supporting its potential role as a sensitive marker of mucosal healing.

## Graphical abstract

**Graphical Abstract:**
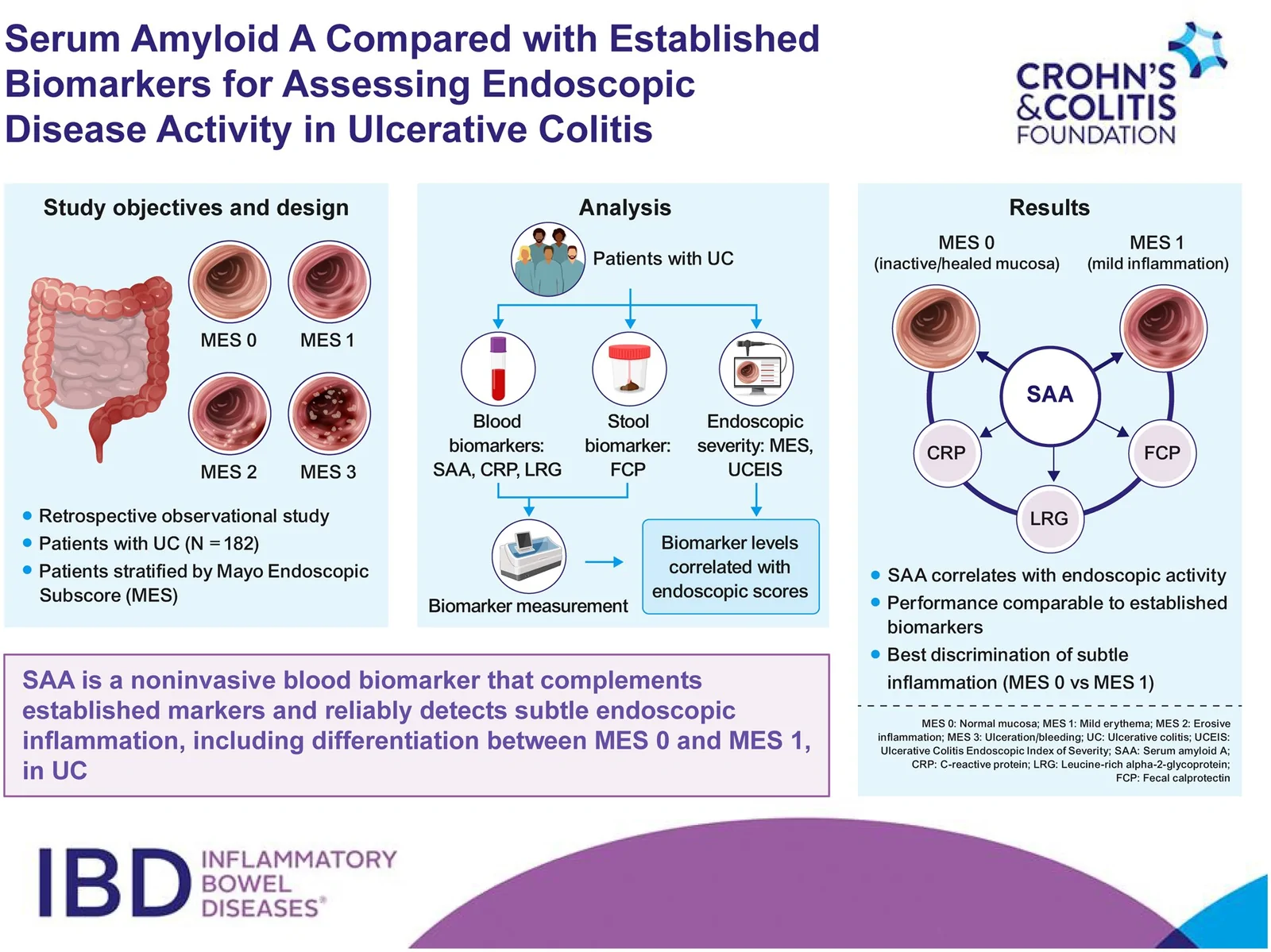

Key Messages **What is already known?** 

- Endoscopic mucosal healing is a central treatment goal in ulcerative colitis, but repeated colonoscopy is invasive and impractical for routine monitoring.
- Established biomarkers such as CRP, LRG, and fecal calprotectin correlate with endoscopic activity, but each has limitations, particularly in detecting subtle inflammation near mucosal healing.
- Distinguishing between MES 0 and MES 1 is clinically important but remains challenging using noninvasive markers.

**What is new here?** 

- Serum amyloid A (SAA) shows strong correlations with endoscopic severity, clinical indices, and established biomarkers in UC.
- SAA outperforms CRP and LRG and is comparable to fecal calprotectin in identifying active inflammation and shows the best performance among tested biomarkers for distinguishing MES 0 from MES 1.
- SAA demonstrates a wide dynamic range and sensitivity that may reflect IL-1β–driven microinflammation not fully captured by conventional biomarkers.

**How can this study help patient care?** 

- SAA may provide a reliable, blood-based tool to detect subtle residual inflammation and confirm complete mucosal healing without repeated colonoscopy.
- Incorporating SAA into routine monitoring could improve treat-to-target strategies and allow more individualized disease assessment in UC.
- Combined use of SAA with existing biomarkers may enhance diagnostic accuracy and support personalized, noninvasive disease monitoring.

## Introduction

Ulcerative colitis (UC) is a chronic, relapsing inflammatory disorder of unknown etiology that imposes a substantial clinical and societal burden.^1^ Contemporary management extends beyond symptom control to include endoscopic healing, sustained quality of life, and prevention of long-term disability.^2^ In particular, complete mucosal healing, defined as a Mayo Endoscopic Subscore (MES) of 0, has emerged as a key therapeutic target within the International Organization for the Study of Inflammatory Bowel Diseases (IOIBD) treat-to-target strategy, given its association with improved long-term outcomes.^2^ Although endoscopy remains the reference standard for assessing mucosal inflammation and healing,^3^^,^^4^ its invasive nature and logistical burden limit its feasibility for frequent monitoring in routine clinical practice. Therefore, the demand for objective and readily measurable indicators of inflammatory activity has intensified. Several biomarkers, including C-reactive protein (CRP), leucine-rich α-2-glycoprotein (LRG), and fecal calprotectin (FCP), show meaningful correlations with endoscopic severity,^5–9^ and normalization of these markers is central to treat-to-target approaches.^2^ However, each has limitations. LRG is influenced by extraintestinal inflammation^10^ and shows lower precision than FCP.^7^ FCP, while specific to intestinal inflammation, exhibits wide variability, inconsistent cutoff values,^8^^,^^9^^,^^11^ and practical challenges related to stool sampling.^12^^,^^13^ These limitations underscore the need for additional biomarkers capable of detecting subtle residual inflammation, especially in cases where there is near complete mucosal healing.

Serum amyloid A (SAA) is a highly sensitive acute-phase protein that is markedly upregulated across a wide range of inflammatory conditions, including autoimmune and rheumatic diseases, infections, malignancy, and atherosclerosis.^14^ Wakai et al. demonstrated that SAA correlates with both clinical and endoscopic disease activity and may identify active intestinal inflammation even in patients with normal CRP levels.^15^ These findings suggest that SAA may provide complementary information to conventional serum biomarkers. However, the clinical relevance of SAA in UC, particularly as a surrogate marker of mucosal healing and its relationship with other established biomarkers, remains insufficiently defined.

In this study, we evaluated the potential utility of SAA as an inflammatory biomarker in UC. Specifically, we assessed its ability to reflect endoscopic disease activity, evaluated its usefulness in identifying mucosal healing, and directly compared its diagnostic performance with established biomarkers, including FCP and LRG. This study helps to further clarify the potential role of SAA as a biomarker for endoscopic disease activity and mucosal healing in contemporary UC management.

## Materials and methods

### Patients

This single-center, retrospective observational study included 182 patients with UC who underwent blood and stool testing within 14 days of colonoscopy at a tertiary care hospital between April 2017 and January 2025. Patients were identified through electronic medical records, and the diagnosis of UC was confirmed based on physician assessment and clinical and endoscopic findings in accordance with the Ministry of Health, Labor and Welfare’s guidelines for inflammatory bowel disease (IBD).^1^

Patients were classified into 4 groups according to the MES (MES: 0-3). The MES definitions were as follows: MES 0, normal or inactive mucosa; MES 1, erythema, decreased vascular pattern, and mild friability; MES 2, marked erythema, absent vascular pattern, and erosions; MES 3, spontaneous bleeding or ulceration.^1^ MES was independently assessed by 3 physicians experienced in UC management who were blinded to patients’ clinical information, laboratory data, and the original endoscopic reports. For MES 0, only cases with unanimous agreement among all evaluators were included. For MES 1-3, the most frequently assigned score was adopted, in accordance with the method described by Vezakis A et al.^16^ All assessments were performed using the same scoring system across all patient groups.

Baseline characteristics—including sex, age at colonoscopy, alcohol use, smoking history, disease phenotype, and extraintestinal manifestations (EIMs)—were compared across MES groups. Laboratory and endoscopic parameters obtained at colonoscopy included white blood cell (WBC) count, hemoglobin, platelet count, serum albumin, CRP, LRG, FCP, SAA, Mayo score, partial Mayo score, and the Ulcerative Colitis Endoscopic Index of Severity (UCEIS).^1^ The Mayo score ranges from 0 to 12 and incorporates stool frequency (0-3), rectal bleeding (0-3), physician’s global assessment (0-3), and endoscopic findings (0-3). The partial Mayo score excludes the endoscopic component and ranges from 0 to 9. UCEIS is a validated endoscopic index ranging from 0 to 8 based on vascular pattern (0-2), bleeding (0-3), and erosions/ulcers (0-3), with higher scores indicating more severe inflammation. Serum SAA and CRP levels were measured using latex agglutination immunoassays. LRG concentrations were determined using a latex turbidimetric immunoassay. FCP levels were measured using an enzyme-linked immunosorbent assay (ELISA). According to the manufacturers’ specifications, the reference ranges were <0.14 mg/dL for CRP, <3.0 mg/L for SAA, <16.0 μg/mL for LRG, and <50.0 mg/kg for FCP. Because this study retrospectively collected biomarkers obtained in routine clinical practice, not all biomarkers were measured in every patient. Therefore, analyses of SAA, CRP, LRG, and FCP were performed using the available data for each biomarker, resulting in different sample sizes across analyses.

### Statistical analysis

All statistical analyses were conducted using EZR, a modified version of R Commander that incorporates functions commonly used in biostatistics.^17^ Data distribution normality was evaluated using the Kolmogorov-Smirnov test. Differences among MES groups were assessed using the Kruskal–Wallis test or one-way analysis of variance, as appropriate. Associations between inflammatory biomarkers and clinical variables were examined using Spearman’s rank correlation coefficient. The agreement between endoscopic scores and each biomarker was evaluated using receiver operating characteristics (ROC) curves. Optimal cutoff values for SAA, CRP, LRG, and FCP were determined from ROC curve analyses. For discrimination between MES 0 and MES 1, cutoff values were selected using the minimum distance to the upper-left corner of the ROC curve. For discrimination between MES 0-1 and MES 2-3, cutoff values were selected using the Youden index, which maximizes the sum of sensitivity and specificity. A *P* value of <.05 was considered statistically significant. Subgroup analyses were performed according to biologic-naïve status. To assess relative variability independent of data distribution, the coefficient of variation (CV) was calculated for each variable. Given that biomarker values generally followed a log-normal distribution, CVs were derived using the geometric mean and geometric standard deviation.

## Results

### Patient characteristics and MES-based subgrouping

A total of 182 patients with UC were included. Baseline characteristics are summarized in Table 1. Females comprised 52.5% of the cohort, and the median age at colonoscopy was 45 years. Extensive colitis was the most common disease phenotype. Median values of key inflammatory biomarkers were as follows: WBC count, 6500/µL; CRP, 0.10 mg/dL; LRG, 16.4 µg/mL; FCP, 703.4 mg/kg; and SAA, 4.2 mg/L.

**Table 1:** The baseline characteristics of the patients in UC.

	All (*n* = 182)
**Sex (male/female)**	86 (47.3%)/96 (52.5%)
**Age at colonoscopy median (IQR) (years)**	45 (9-90)
**Alcohol**	42 (23.0%)
**Smoking:**	
** Current**	24 (13.2%)
** Former**	33 (18.1%)
** Never**	95 (52.2%)
** Unknown**	30 (16.5%)
**Type of disease**	
** E1 (proctitis)**	39 (21.4%)
** E2 (left-side colitis)**	34 (18.7%)
** E3 (extensive colitis)**	106 (58.2%)
** Non-classifiable (NC) (atypical or discontinuous distribution)**	3 (1.6%)
**Extraintestinal manifestations^a^**	23 (12.6%)
** Primary sclerosing cholangitis**	4
** Arthralgia**	8
** Uveitis**	0
** Erythema nodosum**	7
** Thrombosis**	4
** Bronchiolitis**	1
** Thyroid**	1
**Mayo score median (IQR)**	4 (1-6)
**Partial Mayo score median (pMayo) (IQR)**	2 (0-4)
** Stool frequency**	1 (0-2)
** Rectal bleeding**	0 (0-1)
** Physician’s global assessment**	1 (0-1)
**UCEIS median (IQR)**	3 (1-4)
** Vascular pattern**	2 (1-2)
** Bleeding**	0 (0-1)
** Erosions and ulcers**	1 (0-1)
**Intestinal infection**	12 (6.6%)
** CMV reactivation**	9 (4.9%)
** Bacterial infection not CMV reactivation**	3 (1.6%)
**White blood cell (WBC) median (IQR)(/µL)**	6500 (5200-7925)
**Hemoglobin median (IQR) (g/dL)**	13.5 (12-14.5)
**Platelet median (IQR) (×10^4^/μL)**	27.9 (23.4-33.5)
**Albumin median (IQR) (g/dL)**	4.2 (3.8-4.4)
**CRP median (IQR) (mg/dL)**	0.1 (0.03-0.5)
**LRG median (IQR) (µg/mL)**	16.4 (11.2-23.8)
**Fecal calprotectin median (FCP) (IQR) (mg/kg)**	703.4 (200.3-2229.2)
**SAA median (IQR) (mg/L)**	4.2 (2.75-11.4)
**Treatment^b^**	
** 5-ASA**	143
** Steroid (Oral or IV)**	23
** Steroid (enema)**	23
** Immunomodulators**	53
** Infliximab**	10
** Adalimumab**	13
** Golimumab**	2
** Vedolizumab**	4
** Carotegrast methyl**	1
** Ustekinumab**	2
** Risankizumab**	2
** Mirikizumab**	0
** Tofacitinib**	8
** Filgotinib**	2
** Upadacitinib**	3
** Tacrolimus**	3

a Two patients have multiple EIMs.

b Current treatment at the time of endoscopic assessment.

Characteristics stratified by MES are presented in Table S1. Patient distribution was as follows: MES 0, *n* = 19; MES 1, *n* = 87; MES 2, *n* = 60; and MES 3, *n* = 16. All inflammatory biomarkers were significantly associated with MES.

### Association of SAA with clinical disease activity and other biomarkers

Serum SAA levels were evaluated in relation to clinical disease activity and established inflammatory biomarkers, including WBC count, CRP, LRG, and FCP (Table S2). Serum SAA showed positive correlations with LRG, CRP, and FCP, with particularly strong correlations observed for LRG (*r* = 0.752) and CRP (*r* = 0.717). Table S2 summarizes the correlations between SAA and clinical as well as endoscopic findings. SAA levels correlated not only with MES but also with the Mayo score, partial Mayo score, and UCEIS. Serum SAA was also significantly associated with stool frequency, rectal bleeding, and the physician’s global assessment components of the partial Mayo score (all *P* < .01). In addition, SAA correlated with the vascular pattern, bleeding, and erosion or ulceration components of the UCEIS (all *P* < .05).

### Variation range (CV) for each biomarker

To compare biomarker variability independent of scale or reference ranges, the CV was calculated for each inflammatory marker. Because biomarkers generally followed log-normal distributions, CVs were derived using geometric means and geometric standard deviations (Figures S1 and S2). Accordingly, the CV was used to robustly quantify variability across markers (Table 2). LRG exhibited consistently low CVs across MES 0, MES 1, and the combined MES 0-1 categories, indicating minimal variability. The CV of SAA was comparable to that of LRG in MES 0 but increased from MES 1 onward. In contrast, FCP and CRP exhibited relatively large CVs across all MES categories, including MES 0.

**Table 2:** Coefficient of variation (CV) of biomarkers after log transformation across MES categories.

	MES 0	MES 1	MES 2	MES 3	All
**(A) Individual MES categories (MES 0, 1, 2, and 3)**					
**WBC (/µL): Mean [95% CI]** **CV (%)**	5110 [4586-5693] 24.4	6148 [5769-6552] 30.6	7322 [6745-7949] 33.3	6457 [4975-8379] 57.2	6419 [6104-6750] 35.4
**CRP (mg/dL): Mean [95% CI]** **CV (%)**	0.03 [0.02-0.07] 181	0.07 [0.05-0.10] 281	0.22 [0.15-0.32] 297	0.76 [0.31-1.85] 514	0.12 [0.09-0.15] 410
**LRG (µg/mL): Mean [95% CI]** **CV (%)**	11.7 [8.03-17.3] 34.8	13.1 [11.7-14.6] 30.6	22.1 [17.3-28.3] 55.5	44.1 [36.5-53.29] 21.7	17.1 [14.8-19.6] 57.6
**FCP (mg/kg): Mean [95% CI]** **CV (%)**	508.1[130.6-1976.3] 179	348 [170-711] 390	1768.3 [790.4-3956.6] 209	9688 [-] -	629.1 [356.9-1108.8] 420
**SAA (mg/L): Mean [95% CI]** **CV (%)**	2.53 [1.97-3.26] 34.9	3.85 [3.12-4.75] 88.9	8.78 [6.3-12.2] 143	42.4 [17.9-100.3] 303	6.59 [5.21-8.26] 188

	MES 0-1	MES 2-3	MES 1-3
**(B) Combined MES categories (MES 0-1, 2-3, and 1-3)**			
**WBC (/µL): Mean [95% CI]** **CV (%)**	5943 [5613-6293] 30.4	7131 [6551-7762] 39.1	6594 [6251-6955] 35.6
**CRP (mg/dL): Mean [95% CI]** **CV (%)**	0.07 [0.05-0.09] 269	0.28 [0.19-0.41] 376	0.14 [0.11-0.18] 410
**LRG (µg/mL): Mean [95% CI]** **CV (%)**	12.9 [11.7-14.3] 30.5	25.9 [20.6-32.5] 59	17.5 [15.1-20.2] 58
**FCP (mg/kg): Mean [95% CI]** **CV (%)**	364.9 [192.3-692.8] 346	2064.1 [937.8-4543] 222	641.9 [348.4-1182.7] 463
**SAA(mg/L): Mean [95% CI]** **CV (%)**	3.66 [3.03-4.42] 84.8	12.7 [8.79-18.4] 226	6.99 [5.49-8.91] 192

CVs were calculated using the geometric mean and geometric standard deviation, assuming a log-normal distribution.

### Prediction of MES using each biomarker

**Figure 1:**
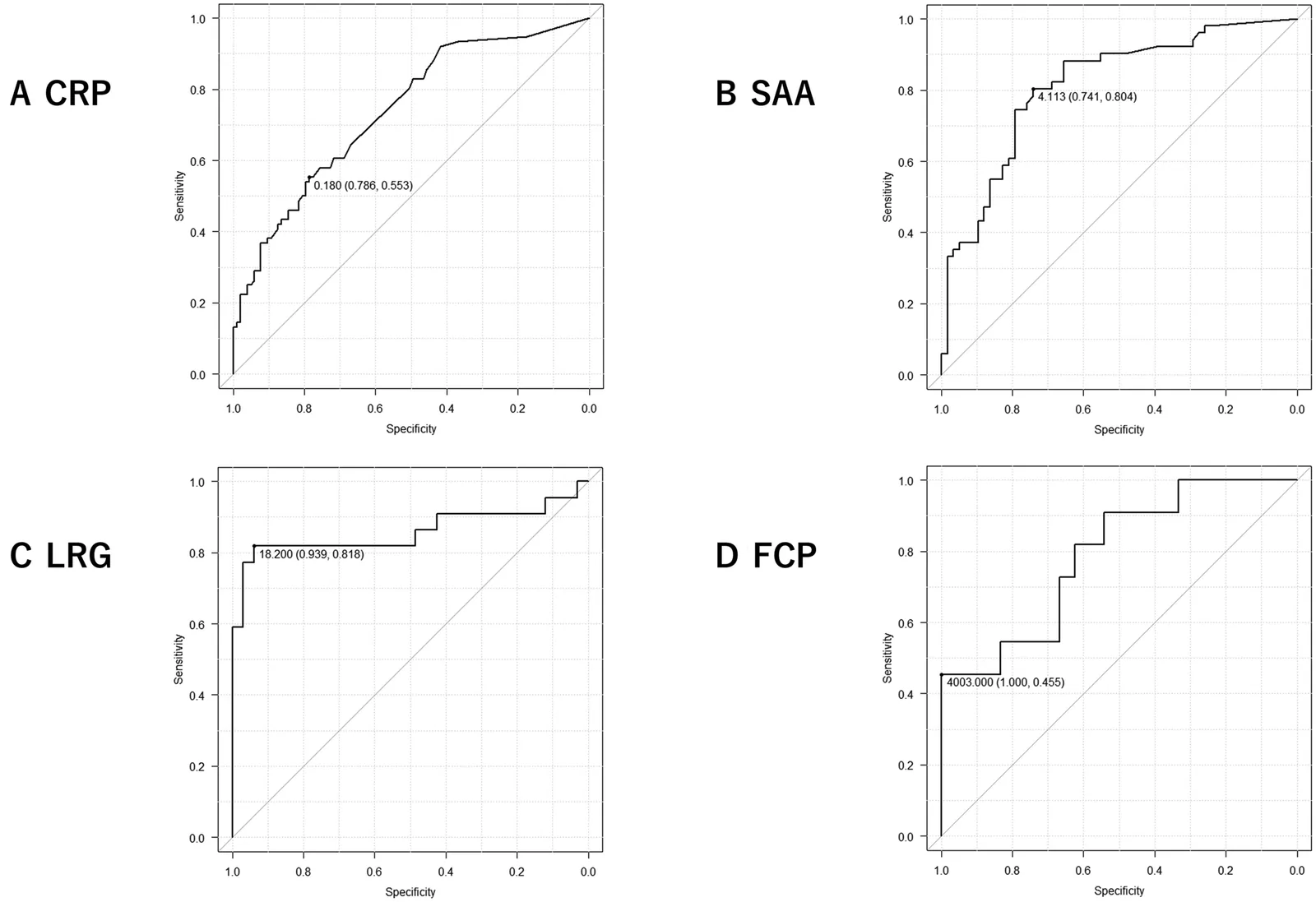
Receiver operating characteristic curve (ROC) results for predicting MES 0-1 and MES 2-3. The panels show CRP (A), SAA (B), LRG (C), and FCP (D).

ROC curve analysis were performed to evaluate biomarker performance for predicting mucosal healing and active inflammation. For active endoscopic inflammation, defined as MES ≥ 2, all biomarkers showed meaningful discriminative ability (Table 3 and Figure 1). LRG demonstrated the strongest performance, with an AUC of 0.858 (95% CI, 0.731-0.986), sensitivity of 0.818, and specificity of 0.939. Optimal cutoff values were 0.18 mg/dL for CRP, 18.2 µg/mL for LRG, 4003 mg/kg for FCP, and 4.1 mg/L for SAA.

**Table 3:** Receiver operating characteristic curve (ROC) results for predicting MES 0-1 and MES 2-3.

	Cut off value	AUC [95%CI]	Sensitivity	Specificity
**CRP**	0.18 (mg/dL)	0.738 [0.665-0.81]	0.553	0.786
**LRG**	18.2 (µg/mL)	0.858 [0.731-0.986]	0.818	0.939
**FCP**	4003 (mg/kg)	0.788 [0.624-0.952]	0.455	1.000
**SAA**	4.1 (mg/L)	0.813 [0.731-0.894]	0.804	0.741

**Figure 2:**
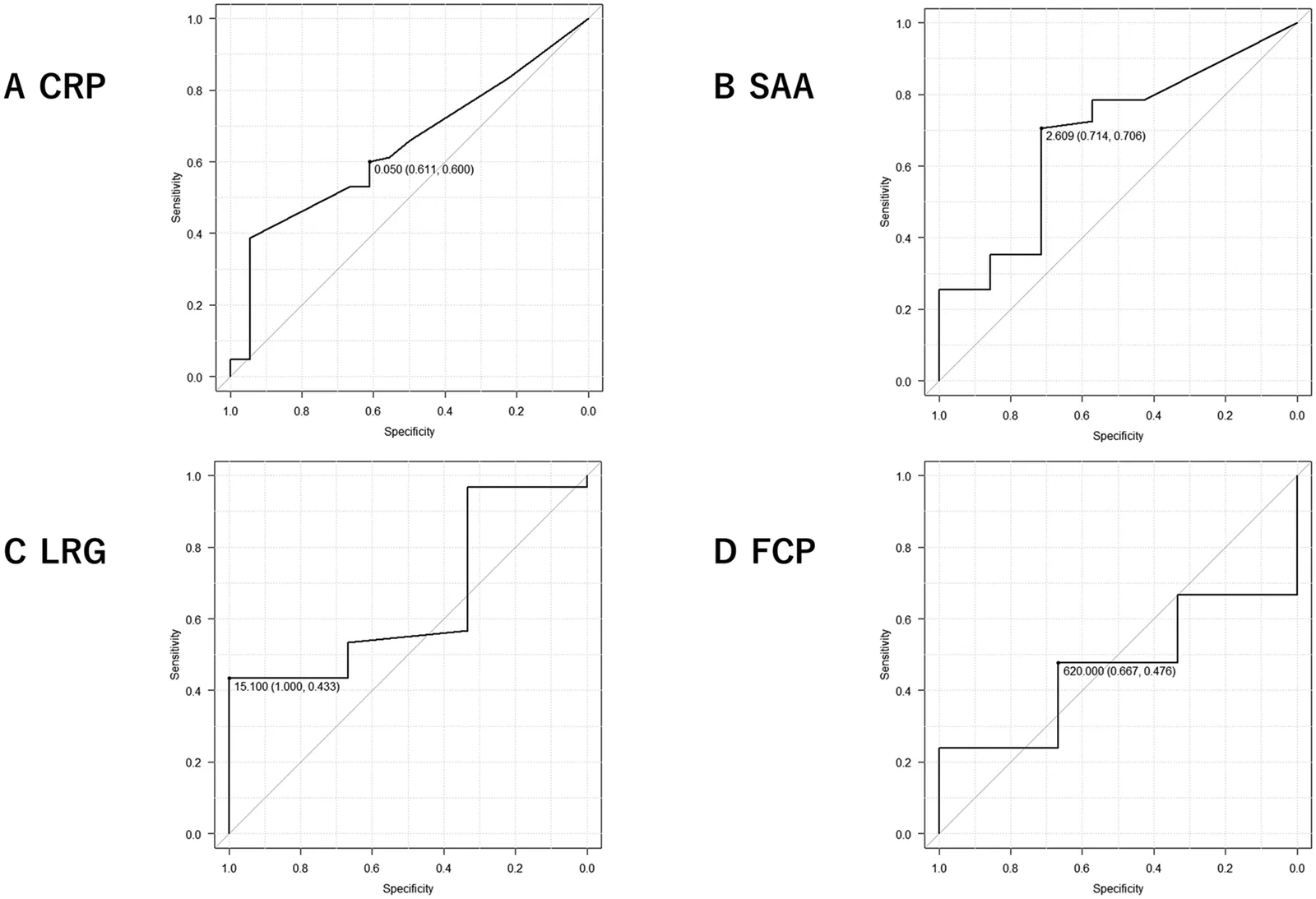
Receiver operating characteristic curve (ROC) results for predicting MES 0 and MES 1. The panels show CRP (A), SAA (B), LRG (C), and FCP (D).

Biomarker performance for differentiating MES 0 from MES 1 is shown in Table 4 and Figure 2. Although AUC values declined across all biomarkers compared with the MES ≥ 2 analysis, SAA demonstrated the highest performance for this distinction, with an AUC of 0.683 (95% CI, 0.470-0.897), sensitivity of 0.706, and specificity of 0.714. Corresponding cutoff values were 0.05 mg/dL for CRP, 15.1 µg/mL for LRG, 620 mg/kg for FCP, and 2.6 mg/L for SAA.

**Table 4:** ROC results for predicting MES 0 and MES 1.

	Cut off value	AUC [95% CI]	Sensitivity	Specificity
**CRP**	0.05 (mg/dL)	0.64 [0.514-0.766]	0.600	0.611
**LRG**	15.1 (µg/mL)	0.65 [0.311-0.989]	0.433	1.000
**FCP**	620 (mg/kg)	0.46 [0.163-0.758]	0.476	0.667
**SAA**	2.6 (mg/L)	0.683 [0.47-0.897]	0.706	0.714

Subgroup analyses in biologic-naïve patients are presented in Table S3 and Figure S3. In this subgroup, AUC values for CRP, LRG, and FCP increased, whereas the AUC for SAA decreased.

Finally, diagnostic accuracy was assessed using the optimal cutoff values shown in Table 4. MES 0 was predicted when both biomarkers were below the cutoff; if either exceeded the cutoff, the predicted score was MES 1. Under this framework, SAA demonstrated the highest standalone diagnostic accuracy. Moreover, combining SAA with LRG or FCP improved overall performance (Table 5).

**Table 5:** Accuracy of each biomarker for distinguishing between MES 0 and MES 1.

	Cut off value	Accuracy	+SAA	+LRG	+FCP
**SAA**	2.6 (mg/L)	0.707 (*n* = 58)	–	0.667 (*n* = 30)	0.688 (*n* = 16)
**LRG**	15.1 (µg/mL)	0.485 (*n* = 33)	0.667 (*n* = 30)	–	0.643 (*n* = 14)
**FCP**	620 (mg/kg)	0.5 (*n* = 24)	0.688 (*n* = 16)	0.643 (*n* = 14)	–

*n* varies according to biomarker availability.

## Discussion

This study suggests that SAA is a clinically relevant biomarker of inflammatory activity in patients with UC. Notably, SAA distinguished MES 0 from MES 1 with greater accuracy than LRG and CRP, a distinction that remains among the most clinically challenging aspects of endoscopic assessment. SAA also correlated strongly with UCEIS and clinical indices, indicating robust reflection of both mucosal and systemic inflammation.

The biological characteristics of SAA support its potential role in this context. Unlike CRP, which is primarily IL-6 dependent, SAA is induced synergistically by IL-6 and IL-1β^18^^,^^19^ and can amplify inflammation through NLRP3 inflammasome activation.^20^^,^^21^ This cytokine responsiveness results in rapid and marked increases, up to 1000-fold, during inflammatory states. Importantly, IL-1β is one of the central cytokines elevated in UC mucosa,^22–25^ and SAA may capture inflammatory pathways not fully reflected by traditional biomarkers. Consistent with this mechanism, SAA demonstrated a wide dynamic range and variability within MES 0 and MES 1, suggesting sensitivity to microinflammatory activity beyond standard endoscopic resolution.

These mechanistic considerations are strongly supported by recent post hoc findings from the SELECTION trial,^26^ which collectively underscore the novelty and clinical potential of SAA in UC: (1) SAA correlated with endoscopic, clinical, and histological inflammation; (2) early reductions in SAA predicted subsequent clinical outcomes; and (3) SAA remained informative in biologic-experienced patients, highlighting its robustness across therapeutic contexts. Collectively, these findings suggest that SAA may represent a valuable biomarker for assessing endoscopic disease activity and detecting subtle residual inflammation in patients with UC. Further prospective studies are needed to determine its clinical utility for longitudinal monitoring and evaluation of treatment response.

In the present study, SAA showed high diagnostic accuracy for identifying MES 0 and enhanced performance when combined with LRG or FCP. Although the accuracy of SAA-based combinations was comparable to that of LRG plus FCP, SAA appeared to provide complementary information. Given interindividual variability in inflammatory profiles among patients with UC,^27^ further studies are required to clarify whether SAA can be universally applied across all clinical contexts. Nevertheless, SAA appears to provide complementary information to existing biomarkers such as FCP and LRG, suggesting comparable clinical utility. Thus, SAA may represent a useful complementary biomarker that could enhance multimarker approaches for evaluating disease activity in UC. It is noteworthy that the optimal FCP cutoff values identified in the present study differed from those previously reported for endoscopic remission in UC.^2^ Given that these thresholds were derived from ROC analyses within a retrospective single-center cohort and optimized for specific endoscopic comparisons, they should be interpreted as cohort-specific findings. External validation should be performed before applying these thresholds more broadly in clinical practice.

This study has several limitations. First, it was a retrospective single-center design with a cohort where many patients had relatively mild disease activity and limited exposure to advanced therapies, this may limit its generalizability. Second, fecal samples were self-collected, and although obtained within 14 days of colonoscopy, sampling timing was not strictly standardized, which may have affected FCP measurements. Third, combined biomarker analyses were restricted to cases with concurrent measurements, resulting in smaller sample sizes and potentially influencing diagnostic accuracy. Fourth, the study focused primarily on endoscopic outcomes and did not assess histologic activity, clinical outcomes, or long-term prognosis. Finally, the lack of serial biomarker measurements precluded evaluation of temporal changes in SAA levels and their association with treatment response. Prospective multicenter studies incorporating histological and clinical outcomes are warranted to further clarify the role of SAA in UC management.

In conclusion, SAA performs comparably to established biomarkers for assessing inflammation in patients with UC and showed potential utility in distinguishing MES 0 from MES 1. SAA may serve as a complementary biomarker for detecting subtle mucosal inflammation. Future prospective studies are needed to define the clinical utility of SAA in patients with minimal residual disease activity and to determine whether its integration into multimarker strategies enhances disease assessment beyond currently available biomarkers.

## Supplementary Material

otag097_Supplementary_Data

## Data Availability

The data underlying this article are not publicly available due to privacy and ethical restrictions but are available from the corresponding author on reasonable request.
