# Supplementary material for: Serum amyloid a as a high-resolution indicator of mucosal healing in ulcerative colitis
Source: Crohns Colitis 360. 2026 Sep 1;8(3):otag097. doi: 10.1093/crocol/otag097 (PMC13570589; doi:10.1093/crocol/otag097)
Supplement: Supplementary file 1 [file otag097_supplementary_data.zip › SAA_table(supplementary)_revised.docx]

Supplementary Table S1. The baseline characteristics of patients in the MES 0, 1, 2, and 3 groups.

| Group | MES 0 (n=19) | MES 1 (n=87) | MES 2 (n=60) | MES 3 (n=16) | P value |
| --- | --- | --- | --- | --- | --- |
| Sex (male/female) | 12 (63.2%)/7 (36.8%) | 40 (46.0%)/47 (54.0%) | 27 (45%)/33 (55%) | 7 (43.8%)/9 (56.2%) | 0.535 |
| Age at colonoscopy median (IQR) (years) | 54 (24-75) | 45 (9-83) | 45.5 (19-83) | 41.5 (17-90) | 0.177 |
| Alcohol | 6 (31.6%) | 18 (20.7%) | 14 (23.3%) | 4 (25.0%) | 0.629 |
| Smoking:  Current  Former  Never  Unknown | 1 (5.2%)  6 (31.6%)  8 (42.1%)  4 (21.1%) | 13 (14.9%)  13 (14.9%)  47 (54.0%)  14 (16.0%) | 8 (13.3%)  11 (18.3%)  34 (56.7%)  7 (11.7%) | 2 (12.5%)  3 (18.8%)  6 (37.5%)  5 (31.2%) | 0.569 |
| Type of disease:  E1 (proctitis)  E2 (left-side colitis)  E3 (extensive colitis)  NC (atypical or discontinuous distribution) | 8 (42.1%)  3 (15.8%)  8 (42.1%)  0 (0%) | 22 (25.3%)  14 (16.1%)  48 (55.2%)  3 (3.4%) | 6 (10.0%)  15 (25.0%)  39 (65.0%)  0 (0%) | 3 (18.7%)  2 (12.5%)  11 (68.8%)  0 (0%) | 0.087 |
| Extraintestinal manifestations*  - primary sclerosing cholangitis  - arthralgia  - uveitis  - erythema nodosum  - thrombosis  - bronchiolitis  -tyroid | 1 (5.2%)  0  1  0  0  0  0  0 | 13 (14.9%)  4  5  0  3  2  1  0 | 5 (8.3%)  0  2  0  2  1  0  0 | 4 (25%)  0  0  0  2  1  0  1 | 0.214 |
| Mayo score median (IQR) | 0 (0-1) | 2 (1-3) | 6 (5-7) | 9 (7-9) | <0.01 |
| pMayo score median (IQR)  -Stool Frequency  -Rectal Bleeding  -Physician’s Global Assessment | 0 (0-0.3)  0 (0-0.3)  0 (0-0)  0 (0-0) | 1 (0-3)  0 (0-1)  0 (0-0)  0 (0-1) | 4 (3-5)  2 (1-3)  1 (0.3-1)  1 (1-2) | 6 (4-6)  3 (2-3)  1 (1-1.8)  2 (1-2) | <0.01 |
| UCEIS median (IQR)  -Vascular pattern  -Bleeding  -Erosions and ulcers | 0 (0-0.5)  0 (0-1)  0 (0-0)  0 (0-0) | 2 (1-2.5)  1 (1-2)  0 (0-0)  0 (0-1) | 4 (3-5)  2 (2-2)  1 (0-1)  1 (1-2) | 5.5 (5-6)  2 (2-2)  1.5 (1-2)  2 (2-2) | <0.01 |
| intestinal infection  -CMV reactivation  - bacterial infection not CMV reactivation | 0  0  0 | 1 (1.2%)  1 (1.2%)  0 | 8 (13.3%)  6 (10%)  2 (3.3%) | 3 (18.7%)  2 (12.5%)  1 | <0.01 |
| WBC median (IQR)(/µL) | 4,900 (4,600-6,000) | 6,200 (5,200-8,200) | 7,500 (5,600-8,925) | 7,500 (6,175-8,200) | <0.01 |
| Hemoglobin median (IQR) (g/dL) | 14 (13.1-14.8) | 13.6 (12.2-14.9) | 13.1 (11.7-14.1) | 12.0 (10.3-13.1) | <0.01 |
| Platelet median (IQR) (×10^4^/μL) | 25.4 (22.4-27.1) | 27 (23.2-30.8) | 31.1 (24.9-36.6) | 37.6 (28.5-40.6) | <0.01 |
| Albumin median (IQR) (g/dL) | 4.3 (4.2-4.6) | 4.3 (4.1-4.5) | 3.8 (3.6-4.2) | 3.4 (3.0-3.7) | <0.01 |
| CRP median (IQR) (mg/dL) | 0.03 (0.02-0.10) | 0.1 (0.02-0.17） | 0.17 (0.1-0.73） | 0.95 (0.2-3.3） | <0.01 |
| LRG median (IQR) (µg/mL) | 13.6 (10.8-14.3) | 14.1 (10.7-16.7) | 23.5 (18.2-31.3) | 47.7 (39.9-50.4) | <0.01 |
| FCP median (IQR) (mg/kg) | 472.5 (315.7-1109.8) | 442 (92-1668) | 1745 (803.7-5076) | 9688 | 0.04 |
| SAA median (IQR) (mg/L) | 2.05 (2-3.1) | 3 (2.4-4.68) | 7.3 (3.9-13.3) | 78.7 (7.8-115.1) | 0.02 |
| Treatment**:  5-ASA  Steroid (Oral or IV)  Steroid (enema)  Immunomodulators  Infliximab  Adalimumab  Golimumab  Vedolizumab  Carotegrast Methyl  Ustekinumab  Risankizumab  Mirikizumab  Tofacitinib  Filgotinib  Upadacitinib  Tacrolimus | 18  1  1  5  0  1  0  0  0  0  1  0  1  0  0  1 | 67  6  9  24  6  4  1  2  0  1  1  0  4  2  2  1 | 45  13  10  18  4  5  1  2  1  1  0  0  2  0  0  1 | 13  3  3  6  0  3  0  0  0  0  0  0  1  0  1  0 |  |

*some patients have multiple EIMs

**Current treatment at the time of endoscopic assessment

Supplementary Table S2. Correlations of SAA with clinical and endoscopic disease activity

|  | r | P value |
| --- | --- | --- |
| Mayo score | 0.51 | <0.01 |
| MES | 0.488 | <0.01 |
| pMayo score  -Stool Frequency  -Rectal Bleeding  -Physician’s Global Assessment | 0.463  0.463  0.298  0.431 | <0.01  <0.01  <0.01  <0.01 |
| UCEIS  -Vascular pattern  -Bleeding  -Erosions and ulcers | 0.483  0.231  0.469  0.463 | <0.01  0.01  <0.01  <0.01 |

Supplementary Table S3. ROC results for predicting MES 0 and MES 1 in bio-naïve (n=79).

|  | Cut off value | AUC [95%CI] | Sensitivity | Specificity |
| --- | --- | --- | --- | --- |
| CRP | 0.04 (mg/dL) | 0.675 [0.532-0.819] | 0.677 | 0.6 |
| LRG | 15.1 (µg/mL) | 0.675 [0.361-0.989] | 0.500 | 1.000 |
| FCP | 358 (mg/kg) | 0.5 [0.0626-0.937] | 0.692 | 0.5 |
| SAA | 2.6 (mg/L) | 0.658 [0.409-0.906] | 0.730 | 0.667 |

Supplementary Table S1. The baseline characteristics of patients in the MES 0, 1, 2, and 3 groups.

Supplementary Table S2. Correlations of SAA with clinical and endoscopic disease activity.

Supplementary Table S3. ROC results for predicting MES 0 and MES 1 in bio-naïve (n=79).
