# Supplementary material for: Serum amyloid a as a high-resolution indicator of mucosal healing in ulcerative colitis
Source: Crohns Colitis 360. 2026 Sep 1;8(3):otag097. doi: 10.1093/crocol/otag097 (PMC13570589; doi:10.1093/crocol/otag097)
Supplement: Supplementary file 1 [file otag097_supplementary_data.zip › Supplementary Figure legends.docx]

**Figure legends (Supplementary)**

Supplementary Figure S1: The normality of each biomarker was assessed using the Kolmogorov-Smirnov test. The panels show white blood cell (WBC) count (A), C-reactive protein (CRP) (B), leucine-rich alpha-2-glycoprotein (LRG) (C), fecal calprotectin (FCP) (D), and serum amyloid A (SAA) (E). An asterisk (*) indicates *P* < 0.001.

Supplementary Figure S2: The normality of each biomarker after natural logarithmic transformation was assessed using the Kolmogorov-Smirnov test. The panels show WBC count (A), CRP (B), LRG (C), FCP (D), and SAA (E). An asterisk (*) indicates *P* < 0.05.

Supplementary Figure S3: Receiver operating characteristic curve (ROC) results for predicting MES 0 and MES 1 in bio-naïve (n=79). The panels show CRP (A), SAA (B), LRG (C), and FCP (D).
